# Assessing Lysosomal Disorders in the NGS Era: Identification of Novel Rare Variants

**DOI:** 10.3390/ijms21176355

**Published:** 2020-09-01

**Authors:** Marisa Encarnação, Maria Francisca Coutinho, Lisbeth Silva, Diogo Ribeiro, Souad Ouesleti, Teresa Campos, Helena Santos, Esmeralda Martins, Maria Teresa Cardoso, Laura Vilarinho, Sandra Alves

**Affiliations:** 1 Research and Development Unit, Human Genetics Department, National Institute of Health Doutor Ricardo Jorge, 4000-055 Porto, Portugal; marisa.encarnacao@insa.min-saude.pt (M.E.); francisca.coutinho@insa.min-saude.pt (M.F.C.); lisbeth.silva@insa.min-saude.pt (L.S.); diogo.ribeiro@insa.min-saude.pt (D.R.); laura.vilarinho@insa.min-saude.pt (L.V.); 2 Newborn Screening, Metabolism & Genetics Unit, Human Genetics Department, National Institute of Health Doutor Ricardo Jorge, 4000-055 Porto, Portugal; 3 Center for the Study of Animal Science, CECA-ICETA, University of Porto, 4051-401 Porto, Portugal; 4 Biochemical Service, CHU Farhat Hached, 4000 Sousse, Tunisia; souad_wb@yahoo.fr; 5 Reference Center for Inherited Metabolic Disorders, University Hospital Centre S. João, 4202-451 Porto, Portugal; teresaalmeidacampos@gmail.com (T.C.); mtcpteresa@gmail.com (M.T.C.); 6 Department of Pediatrics, Hospital Centre, EPE, 4434-502 V.N. Gaia, Portugal; maria.helena.santos@CHVNG.MIN-SAUDE.PT; 7 Oporto Hospital Centre, University of Porto, 4099-001 Porto, Portugal; esmeralda.g.martins@gmail.com

**Keywords:** lysosomal storage diseases (LSDs), diagnostics odyssey, next-generation sequencing (NGS), molecular genetic testing (MGT), bioinformatics analysis, GM2 Gangliosidosis, CLN7, *GM2A* gene

## Abstract

Lysosomal storage diseases (LSDs) are a heterogeneous group of genetic disorders with variable degrees of severity and a broad phenotypic spectrum, which may overlap with a number of other conditions. While individually rare, as a group LSDs affect a significant number of patients, placing an important burden on affected individuals and their families but also on national health care systems worldwide. Here, we present our results on the use of an in-house customized next-generation sequencing (NGS) panel of genes related to lysosome function as a first-line molecular test for the diagnosis of LSDs. Ultimately, our goal is to provide a fast and effective tool to screen for virtually all LSDs in a single run, thus contributing to decrease the diagnostic odyssey, accelerating the time to diagnosis. Our study enrolled a group of 23 patients with variable degrees of clinical and/or biochemical suspicion of LSD. Briefly, NGS analysis data workflow, followed by segregation analysis allowed the characterization of approximately 41% of the analyzed patients and the identification of 10 different pathogenic variants, underlying nine LSDs. Importantly, four of those variants were novel, and, when applicable, their effect over protein structure was evaluated through in silico analysis. One of the novel pathogenic variants was identified in the *GM2A* gene, which is associated with an ultra-rare (or misdiagnosed) LSD, the AB variant of GM2 Gangliosidosis. Overall, this case series highlights not only the major advantages of NGS-based diagnostic approaches but also, to some extent, its limitations ultimately promoting a reflection on the role of targeted panels as a primary tool for the prompt characterization of LSD patients.

## 1. Introduction

Lysosomal storage diseases (LSDs) are a class of rare, monogenic, inherited metabolic diseases caused by pathogenic variants in proteins critical for lysosomal function [1].

These variants may occur in more than 60 genes, which encode a panoply of proteins directly involved in lysosomal function and homeostasis: soluble acidic hydrolases, integral membrane proteins, activators, transporters, or even nonlysosomal proteins [1,2]. Pathogenic variants in these genes lead to the aberrant processing and degradation of substrates, impaired traffic of lipids and metabolites and progressive primary accumulation of non-degraded or partially degraded molecules inside lysosomes, ultimately resulting in cellular dysfunction and/or death [3]. Patients with LSDs present a debilitating, multisystemic phenotype often associated with early onset neurodegeneration, while others may present only mild neurological symptoms or even be non-neuronopathic. LSDs patients, like other rare disease patients, often face a difficult journey to diagnosis, commonly termed a “diagnostic odyssey,” which frequently involves moving from clinician to clinician, multiple misdiagnosis, unnecessary tests, and even incorrect treatments. For LSDs in particular, this may be partially justified by the considerable clinical overlap and variability [3]. Generally, the route to a definitive diagnosis is based upon the clinical presentation and a panel of laboratory tests, mostly performed on blood and urine. Traditionally, most LSDs cases are diagnosed by demonstrating a deficiency of the activity of a lysosomal enzyme. The panel of enzymes assayed depends on the clinical indications and the nature of any storage products that may be identified in a preliminary screen in urine or plasma [4]. For most laboratories, molecular genetic testing (MGT) is the following step on the algorithm for diagnosis. Genomic sequencing of involved genes is recommended in order to confirm diagnosis and is also the only method that supports safe prenatal diagnosis. Furthermore, it may be the only method allowing for definitive diagnosis for those disorders for which there is no biochemical test (e.g., several forms of neuronal ceroid lipofuscinosis (NCL)) or to clarify the effect of a pseudodeficiency [5].

While being an integral part of virtually every diagnostic algorithm, only recently has MGT started to be used as a primary diagnostic test for LSD. This paradigm shift largely owes to the emergence of rapid, reliable and affordable high-throughput DNA sequencing—next-generation sequencing (NGS). This technology allows for the investigation of variants at genomic scale providing valuable information especially for diseases characterized by obvious genetic and phenotypic heterogeneity [6,7]. For LSDs in particular, targeted panel sequencing and whole exome sequencing (WES) have been successfully applied, namely for the molecular diagnosis of NCLs, MPSs, and GM2 glangliosidoses, among others. Since LSDs are genetically heterogeneous diseases whose symptoms overlap with those of other diseases, NGS-targeted panels may actually be the best choice as first-line molecular tests compared with WES, since are both cheaper and faster/easier to analyze.

Also, taking in consideration that missense variants are the most frequent LSD-causing variants [8,9], the aim of this work was to use a targeted NGS-based workflow for the identification of point variants (missense, nonsense and small indels) in exons and their respective intronic flanking regions in genes involved in lysosomal function. Most of those genes are LSD-related. Nevertheless, we have also included in our panel design a number of genes associated with diseases with symptoms overlapping LSDs and a few others, which have never been associated with disease, even though coding for proteins that assume relevant roles for proper lysosomal function. Altogether, the panel includes a total of 85 genes, and using it, we were able to molecularly characterize nine out of 23 patients with clinical and/or biochemical suspicion of LSD.

## 2. Results

### 2.1. NGS Results

Here, we report 23 patients from different countries (13 Portuguese patients, seven Tunisian, two from India, and one from Cape Verde) with variable degrees of suspicion of LSD depending on their clinical and/or biochemical features.

**Figure 1:**
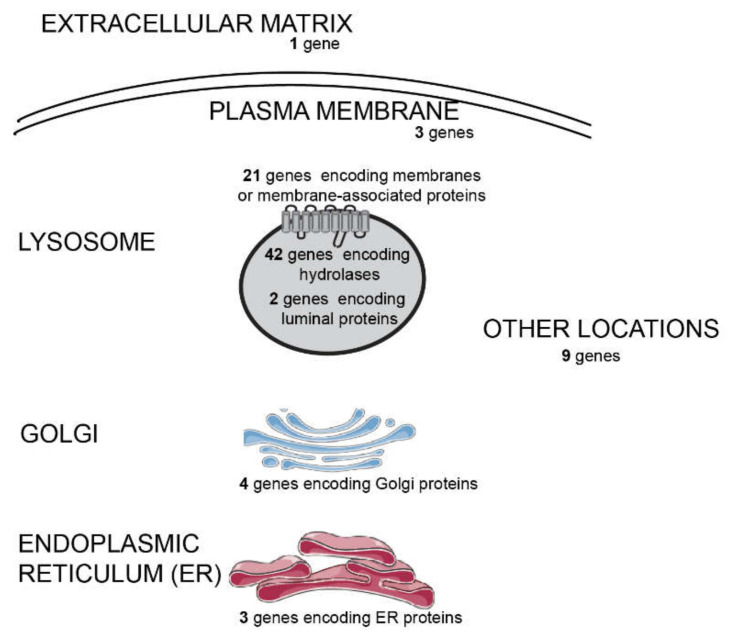
Subcellular distribution of the different gene products whose coding sequences were included in our next generation sequencing (NGS) panel designed to diagnose lysosomal storage diseases (LSDs) and related disorders. The panel is composed by 85 genes, as depicted: 65 genes encoding lysosomal proteins and 20 genes coding for non-lysosomal proteins. Some genes associated to diseases resembling LDSs or genes encoding proteins associated with lysosome-related organelles (LROs) were also covered, in such a way that, from a pathophysiological point of view, the panel includes 54 LSD-related genes, 13 which are disease-causing but non-LSD-related, and 18 not previously associated to disease. The non-LSD-disease-causing genes include three involved in LRO disorders and 10 whose deficiency is known to underlie disorders with overlapping phenotypes.

In order to molecularly characterize them, we used an in-house customized extended NGS targeted panel to screen for LSDs and related diseases, which allows the simultaneous sequencing of 85 genes involved in lysosomal function (Figure 1).

Using our pipeline, which included the use of our customized LSD panel followed by data analysis and Sanger sequencing validation of the identified variants, we were able to provide genetic diagnosis to nine patients with a high clinical suspicion index for LSD (high likelihood based on the presence of LSD hallmark clinical and/or biochemical features).

In summary, we identified four novel pathogenic variants and six previously identified pathogenic variants (Table 1). Yet, from the overall MGT results, we would like to highlight the molecular characterization results of two patients in particular, for whom the application of a targeted NGS screen for LSDs allowed for a fast and reliable diagnosis, which would have been particularly hard and/or time-consuming to achieve using classical MGT approaches. The first case was that of a patient in whom we identified a pathogenic variant in the *GM2A* gene. Pathogenic variants in this gene are associated with an extremely rare variant of gangliosidosis: the GM2A gangliosidosis AB variant. This disorder is clinical and biochemically undistinguishable from Tay–Sachs and Sandhoff diseases [10] and, to the best of our knowledge, only 12 cases have been reported worldwide. The second case refers to a Tunisian patient with a general clinical suspicion of NCL, in whom we identified a pathogenic variant in *MFSD8* gene, which is reported as causative of CLN7, a late infantile NCL. This is particularly relevant since this group of diseases results from pathogenic variants in 1 of 14 different genes [11,12,13,14] that share common clinical and pathologic etiologies [11]. There, the NGS-panel approach provides a faster and effective molecular diagnosis.

### 2.2. Novel Variants

**Figure 2:**
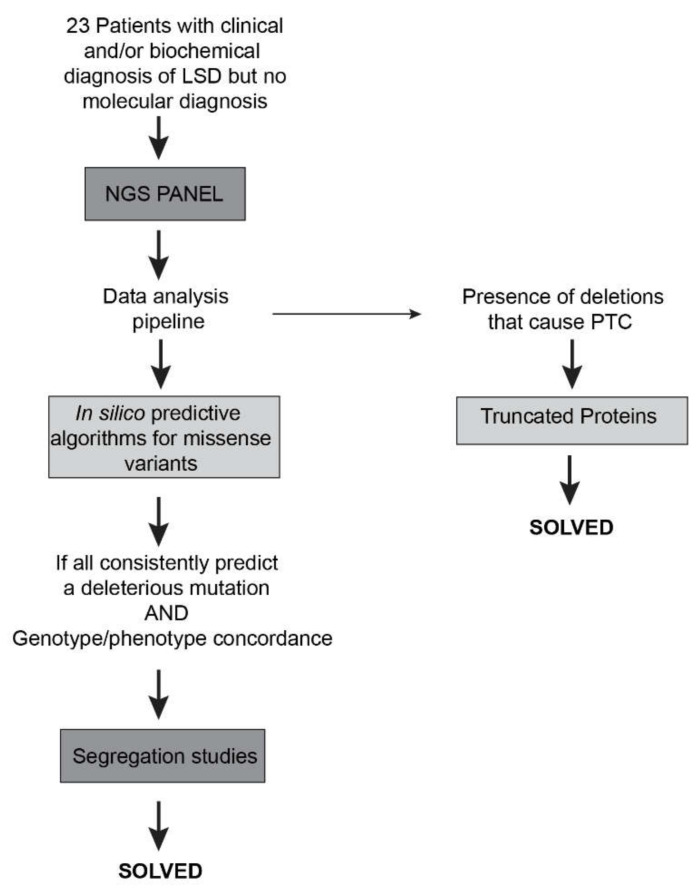
Workflow of the study of the patients and respective molecular characterization The NGS-targeted gene panel allowed the identification of four novel and six previously identified pathogenic variants in a group of 23 patients. PTC = premature termination codon.

To address the pathogenicity of the novel missense variants, we relied on the software wANNOVAR that gives information on the in silico predictions and variants frequency. Whenever a novel variant was predicted as pathogenic, we confirmed its presence by classical sequencing and performed segregation analysis (Figure 2, left side). In general, we might say that the initial in silico predictions were found to be quite accurate, with all the potentially pathogenic variants nicely segregating among the affected families and all downstream assessments further supporting their pathogenicity. This is in agreement with a recent report on the structural basis of Fabry disease, in which the wANNOWAR software was tested for its accuracy in predicting the pathogenicity of several variants (already described and proved to be pathogenic) in the *GLA* gene. In general, the wANNOWAR in silico predictions were in agreement with the pathogenicity of the variants under analysis. Furthermore, the software was also able to correlate the severity of the phenotype to the respective score of the causative variant [24]. Therefore, our results add up to the overall positive track record of wANNOWAR as a powerful tool to address the pathogenic potential of novel variants in LSD-related genes.

On the other hand, whenever we identified novel deletions and/or duplications causing premature termination and/or frameshifts, their predictive molecular consequence were mostly obvious, as they were expected to lead to absent or truncated proteins, which result in a complete loss of functional enzyme/protein (Figure 2, right side). Therefore, their in silico pathogenicity assessment offered no significant challenges.

Over the following sections, we will briefly describe the novel variants identified in this study. In general, we have grouped the variants according to the sub-group of LSDs each one of them underlies.

#### 2.2.1. Shingolipidoses: p.Trp287Arg (GLA Gene), p.Gly104GlyfsTer14 (GM2A Gene) and p.Tyr205Ter (GALC Gene)

**Figure 3:**
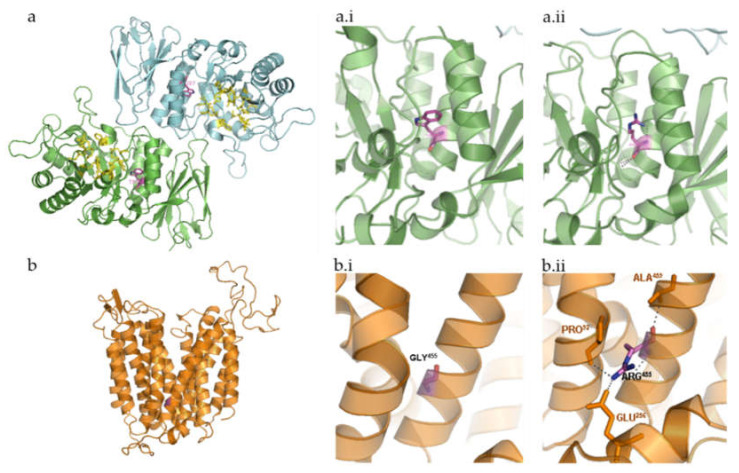
Three-dimensional structure representation of α-galactosidase (GLA) and major facilitator superfamily domain containing 8 (MFSD8) using PyMol. β-strands, helices, and coils indicate the secondary structure elements that form the scaffold for the interacting residues. Relevant side chains that interact with the residues of interest are depicted and labelled. (**a**) Human GLA dimer, shown in ribbon representation (Protein Data Bank accession code = 1R46). Each monomer of the homodimer contains two domains, α(β/α)_8_ barrel containing the active site plus a C-terminal antiparallel β domain. Active site residues are shown in yellow (Trp^47^, Asp^92^, Asp^93^, Tyr^134^, Cys^142^, Lys^168^, Asp^170^, Glu^203^, Leu^206^, Tyr^207^, Arg^227^, Asp^231^, Asp^266^, Met^267^). Relevant side chains that interact with the residue of interest are depicted and labelled. α-galactosidase A dimer, with the active site residues and the affected amino acid highlighted; (**a.i**) wild-type Trp^287^; (**a.ii**)—mutated Arg^287^; (**b**) in silico design of MFSD8 three-dimensional structure based on sequence analysis and subsequent prediction of the secondary structure elements it encodes. The three-dimensional structure depicted does not take into consideration the fact that this is a multi-pass membrane protein. Instead, it refers to the mature protein MFSD8 as it would coil based solely on the intra-molecular interactions. Still, a comparison between this prediction and that of the transmembrane domains further validates the theoretical assumption that MFSD8 contains 12 transmembrane domains, which are formed by α-helices. (**b.i**) Wild-type Gly^455^. (**b.ii**) Mutated Arg^455^.

We identified the variant p.Trp287Arg in a Tunisian patient (patient P2, Table 1) with clinical phenotype of Fabry disease (OMIM#301500). In general, Fabry disease causing-variants are divided into two different types: those that affect the active site of the enzyme α-galactosidase (GLA) and those that adversely affect the stability of the folded protein [25]. Surprisingly, most of the residues affected by Fabry disease point variants do not cluster around the active site but are found to be distributed throughout the hydrophobic core of the protein. In fact, most of the *GLA* disease-causing missense variants produce changes in its hydrophobic core, with 65% of the missense pathogenic variants coding for buried amino acid residues, according to Garman and Garboczi [26]. The new *GLA* missense variant here reported affects one of those buried aminoacids: Trp^287^, right in the hydrophobic core of the protein (Figure 3a). It is expectable that the substitution of tryptophan by arginine disrupts the GLA hydrophobic core. It is also important to stress that there are already three known pathogenicvariants affecting residue Trp^287^: a nonsense variant (p.Trp287Ter [27]) and two missense ones (p.Trp287Gly [28,29]), all of them associated with a severe phenotype [25]. Accordingly, the reported case also presents a classic phenotype (even though no detailed clinical data was provided). Furthermore, the variant was also detected in heterozygosity in the patient’s mother. Moreover, when this patient’s brother was screened for this novel variant, he was also shown to harbor it, in hemizygosity and this boy was also shown to be affected. This is particularly relevant for Fabry disease since this is one of the LSDs for which there is a number of available therapeutic approaches. Furthermore, LSDs have generally been considered as diseases that greatly benefit from an early diagnosis because the available treatments produce better clinical outcomes when started early in life. Thus, any method, which ultimately contributes for a prompt, pre-symptomatic, diagnosis, has added value for these disorders.

**Figure 4:**
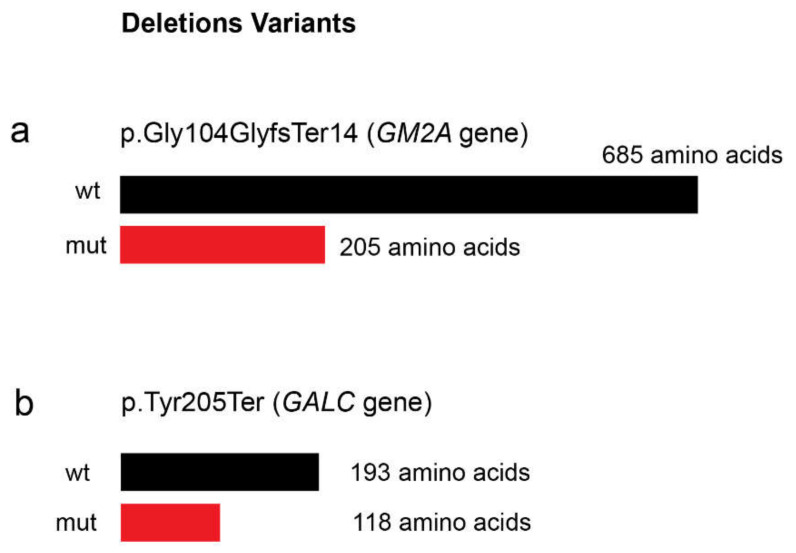
Prevision of the effect of the novel identified deletions ((**a**) c.312del in *GM2A* gene and (**b**) c.613_617del in *GALC* gene) over protein size. wt = non mutated protein; mut = mutated protein.

The second sphingolipidosis that we detected after running our NGS targeted panel on the set of patients here reported was a rare form of Gangliosidosis GM2 (OMIM#272750). We identified a frameshift variant in *GM2A* gene due to a base pair deletion, in a Tunisian patient (patient P7) with a general clinical suspicion of a neurodegenerative LSD. The *GM2A* gene encodes a tiny glycolipid transporter GM2 activator protein (GM2AP), which acts as a substrate specific cofactor for the degradation of GM2 gangliosides by the enzyme β-hexosaminidase [30]. GM2 gangliosides are glycosphingolipids present in the outer layer of mammalian cells. Importantly, these particular gangliosides are significantly enriched on the neuronic surfaces [30]. In normal conditions, glycosphingolipids are catabolized by lysosomal exohydrolases which are unique as they require synthesis and interaction of three-gene products; α- and β-subunits of β-hexosaminidase and presence of the cofactor GM2AP [30]. Hence, deficiency of any of these proteins that are encoded by the *HEXA*, *HEXB*, and *GM2A* genes, respectively, causes excessive intra lysosomal accumulation of GM2 gangliosides and related glycolipids, especially in neuronal cells resulting in GM2 gangliosidosis [31]. To the best of our knowledge, only 12 pathogenic variants have been described in the literature for *GM2A* so far (information collected on HGMD database on 10 July 2020), being an extremely rare variant of GM2 Gangliosidosis. As previously mentioned, this variant is clinically almost indistinguishable of the other two gangliosidoses: Tay–Sachs disease (OMIM #272800) or Sandhoff disease (OMIM #268800) [10]. Therefore, in the case of these diseases, neither the clinical nor the biochemical diagnosis are sufficient to achieve that goal. This further highlights the need for molecular analysis to establish a correct diagnosis, and this NGS analysis allowed that in a quick way. Although a functional study has not been carried, the frameshift variant in the present case is expected to produce a premature stop codon (PTC) (Figure 4a), thus affecting the stability of the mutated protein or its β-hexosaminidase-A binding capabilities or even nonsense mediated decay (NMD). Segregation analysis was also performed and both parents were found to be heterozygous for this deletion, further supporting its causality.

Finally, for the third sphingolipidosis diagnosed in this patient set, we found a pathogenic variant in *GALC* gene in a patient with clinical picture of Krabbe disease (patient P6). The analysis of this novel variant is also more straightforward than that of a missense variant because it also leads to the appearance of a PTC with consequent production of a truncated protein (Figure 4b). *GALC* encodes galactosylceramidase, a lysosomal hydrolase, which is involved in sphingolipid degradation. Currently, more than 70 *GALC* variants are associated with Krabbe disease (information collected on HGMD database—on 10th July 2020). The novel variant here reported is a deletion in the exon 6 of *GALC* gene: c.613_617del. The same variant was found in the mother gDNA in heterozygosity. It is important to notice that we had no access to a sample from the father. Therefore, segregation studies are incomplete for this particular patient. Nevertheless, the overall effect of this 5-nucleotide deletion is mostly obvious, and its classification as a pathogenic variant offers no doubts.

#### 2.2.2. Neuronal Ceroid Lipofuscinoses: p.Gly455Arg (MFSD8 Gene)

The patient P5 harbors pathogenic variant in homozygosity in *MFSD8* gene (p.Gly455Arg)*,* which is associated with the late infantile CLN7. The late infantile CLN7, also known as Turkish variant, is characterized by progressive neurodegeneration with epilepsy, developmental delay, ataxia, speech impairment and vision loss being the most remarkable clinical signs of disease [3]. Based on sequence homology analyses, *MFSD8* encodes for a protein that belongs to the major facilitator superfamily of transporter proteins. It was therefore deduced to act as a lysosomal transporter. Bioinformatic analysis of major facilitator superfamily domain containing 8 (MFSD8) predicted the existence of 12 transmembrane domains with both the N- and C-termini extending into the cytosol [32]. The residue that is involved in the novel variant here reported, Gly^455^, is located inside one of the helical loops that form one of those transmembrane domains. Glycine is a tiny and non-polar aliphatic amino acid with a neutral side chain, which contributes to stabilize the helix. The pathogenic variant here described results in its substitution by arginine, a large residue with a basic polar side chain that disrupts the hydrophobic interior of the helix, thus holding potential to affect membrane insertion (Figure 3b). To the best of our knowledge, there are no more CLN7-causing variants involving this residue. Still, another residue in its surroundings, Thr^458^, which is also involved in the formation and stabilization of the helix that constitutes the 11th transmembrane domain of the MFSD8 protein, has already been found mutated in Romanian patients [12]. That pathogenic variant, p.Thr458Leu, like the one we report here, is associated with a relatively uniform clinical phenotype, the so-called variant late-infantile onset NCL, which is compatible with a complete loss of gene function [33]. Since this group of diseases results from pathogenic variants in one of 14 different genes [11,12,13,14] that share common clinical and pathologic etiology [11], the NGS-panel approach provides a much quicker molecular diagnosis than any classical sequencing approach.

### 2.3. Previously Reported Pathogenic Variants

In addition to the novel pathogenic variants disclosed on the previous sections, a number of known LSD-causing variants have also been readily identified with this extended NGS targeted panel to screen for LSDs and related diseases.

We will briefly list those variants. Whenever considered relevant, their effect over protein structure and/or function will also be further addressed.

#### 2.3.1. Shingolipidoses: p.Leu483Pro (*GBA* Gene)

Apart from the novel variants identified in the *GLA* gene, *GM2A* gene, and *GALC* genes, only one known *GBA* pathogenic variant was detected in Sphingolipidoses-related genes: the p.Leu483Pro in the *GBA* gene (patient P1). This gene encodes for β-glucocerebrosidase (GCase; EC 3.2.1.45), the hydrolase defective in Gaucher disease (GD; MIM# 230800; 230900; 231000). p.Leu483Pro (formerly referred to as L444P [15]) is a well-known GD-causing variant and one of the few for which strong genotype-phenotype correlations have been established. In fact, this pathogenic variant is usually associated with type 3 GD (MIM# 231000), with significant neurovisceral manifestations. The effect of this variant has been addressed by a number of teams and it has already been demonstrated in vitro that this particular amino acidic substitution leads to an unstable protein, with 5.7–9% of wt GCase activity (reviewed in Duarte A.J. et al [34]).

#### 2.3.2. Mucopolysaccharidoses: p.Asp312Asn (*NAGLU* Gene)

Concerning MPS-related genes, we identified the p.Asp312Asn (c.934G>A) missense variant in *NAGLU* gene in homozygosity in a patient (patient P10) with clinical phenotype of MPS III. The *NAGLU* gene encodes α-*N*-acetylglucosaminidase (NAGLU; EC 3.2.1.50), a lysosomal enzyme that specifically degrades heparan sulfate by hydrolysis of terminal N-acetyl-D-glucosamine residues in N-acetyl-α-D-glucosaminides. Defects in this gene result in MPS III B (MIM# 252920) [35] which is characterized by intralysosomal accumulation and urinary excretion of heparan sulfate. The p.Asp312Asn *NAGLU* pathogenic variant has already been reported in two independent studies [18,36]. It was first identified in a French patient [18] in heterozygosity with yet another MPS IIIB-causing variant p.Arg565Gln [37]. While no functional or biochemical studies have been performed on the variant itself at that time, its classification as an MPS IIIB–causing variant offered no doubts, as the patient who harbored it had a previous biochemical diagnosis for the disorder. Later, the same variant was also detected in a second family with intellectual disability (ID) that was enrolled in a homozygosity mapping study through single-nucleotide polymorphism (SNP) genotyping and whole genome sequencing WGS to identify the disease-associated loci and pathogenic and genetic variations [38]. Altogether, these studies support the pathogenicity of the previously reported p.Asp312Asn *NAGLU* missense variant. That assumption was also in accordance with the wANNOVAR in silico predictions here presented, and the variant segregation in both parents. When we assessed the effect of the p.Asp312Asn on the three-dimensional structure of the NAGLU protein, its pathogenicity seemed obvious. In fact, aspartic acid is a negatively charged, polar amino acid. When buried within the protein, aspartic acids are frequently involved in salt bridges, where they pair with a positively charged amino acid to create stabilizing hydrogen bonds that can be important for protein stability. That is the case of Asp^312^, an extremely conserved amino acid, which is buried inside NAGLU (Supplementary Figure S1a), and establishes a strong polar contact with Gln^350^, thus helping to hold together the overall protein structure (Supplementary Figure S1a.i). The p.AspD312Asn variant involves the substitution of that particular aspartic acid residue by one asparagine, which differs only in that it contains an amino group in place of one of the oxygens found in aspartic acid, thus lacking its negative charge (Supplementary Figure S1a.ii).

#### 2.3.3. Glycoproteinoses: c.2402dup (*MAN2B1* Gene)

Even though no novel pathogenic variants have been identified in Glycoproteinoses-related genes in the set of patients here reported, we have detected a known guanine duplication in the 2402 position of the *MAN2B1* gene transcript (c.2402dupG [19]. *MAN2B1* codes for lysosomal α-mannosidase (LAMAN = MAN2B1, EC 3.2.1.24), the enzyme deficient in alpha-mannosidosis (MIM# 248500) [39]. The c.2402dup pathogenic variant, which we have identified in patient P13, had already been reported in 2012, together with 82 other alpha-mannosidosis-associated sequence variants. On the original study, functional analyses were performed for all reported *MAN2B1* missense variants. Nevertheless, like all the other frameshift variants identified in that study, this duplication was readily considered pathogenic for its predicted truncation effect (p.Ser802GlnfsTer129).

#### 2.3.4. Other Enzyme Defects: p.Gly146Arg (*CTSK* Gene)

In addition to the pathogenic variants identified in genes involved in “classical” LSD (those that are grouped in broad categories according to the nature of the substrate), we have also identified a pathogenic variant in the *CTSK* gene, which encodes one of the lysosomal proteases: cathepsin K (CTSK; EC 3.4.22.8). Homozygous or compound heterozygos variants in the *CTSK* gene are known to cause Pyknodysostosis (MIM# 601105) [20]. CTSK is a cysteine protease gene that is highly expressed in osteoclasts. This pattern correlates with the hallmark features of pycnodysostosis, which are deformity of the skull, maxilla, and phalanges; osteosclerosis; and fragility of bone. The missense variant that we have detected in this study (p.Gly146Arg; patient P3) occurs at a CpG dinucleotide and had originally been found in American Hispanic and Moroccan Arab families. Because this missense variant alters the charge of this residue, which resides near the active cysteine, and no member of the papain family has a basic residue in this position, it was predicted to alter cathepsin K activity, thus justifying the associated phenotype [20].

#### 2.3.5. Post-Translational Modification Defects: p.Val191Ile (*GNPTAB* Gene)

In the set of patients assessed in this work, only one pathogenic mutation was detected in Mucolipidoses-related genes: the previously reported p.Val191Ile substitution in the *GNPTAB* gene [17]. This variant was present in homozygosity in patient P4. Segregation analysis showed that both parents were heterozygous for the substitution.

The *GNPTAB* gene encodes the α- and β- subunits of the Golgi-resident GlcNAc-1-phosphotransferase (UDP-GlcNAc; lysosomal enzyme N-acetylglucosamine-1-phosphotransferase; EC 2.7.8.17), an enzyme whose role is crucial for the transport of newly synthesized hydrolases to the lysosome. Variants in the *GNPTAB* gene are associated with Mucolipidosis type II or type III alpha/beta (MIM# 252500 and 252600, respectively), clinical entities that are biochemically characterized by an increase of lysosomal hydrolases activity in the blood patients and present remarkable phenotypic features [40].

Interestingly, the p.Val191Ile was originally reported in a general mutation update study that listed all the ML II and III-causing variants known in 2019. That manuscript provided an overview on 258 and 50 variants in *GNPTAB* and *GNPTG*, respectively, including 58 novel *GNPTAB* and seven novel *GNPTG* variants. The p.Val191Ile was one of the novel variants included in that study. Its effect at RNA and protein level was not tested in vitro but in silico predictors classify it as a variant most likely affecting pre-mRNA splicing [17]. This is in accordance with our own predictions. In fact, using the web server wANNOVAR, the missense variation p.Val191Ile is benign. However, this variant occurs in the last nucleotide of the exon 5, thus removing a consensus splice site. Using the prediction algorithm MaxEntScan, the score for the splicing in the normal sequence is almost two-fold higher than the splicing score in the mutated sequence. Theoretically, whenever the score variation between normal and mutant sequence is above 30%, the variant is expected to create a splice site [41] Aberrant splicing frequently causes a frameshift and the introduction of a PTC. Taking into account this patient had a clinical suspicion of ML II alpha/beta, the most severe ML phenotype, a “benign” homozygous missense mutation would hardly qualify as its underlying genotype. On the other hand, if this variant does impact splicing and protein production, the severe ML II phenotype would be expectable.

## 3. Discussion

While holding some well-known drawbacks, the clinical utility of NGS approaches for MGT is undeniable. Over the last decade, a significant number of teams have developed targeted panels for LSDs, which allowed for the molecular characterization of an even higher number of LSDs patients, ending up several diagnostic odysseys [39,42,43,44,45,46,47,48,49,50,51]

In general, panel design may vary significantly depending on its underlying rationale: for LSDs in particular, some teams have developed small and fast-targeted NGS approaches, which rely on the analysis of a small number of genes selected on the basis of overlapping clinical manifestations [41], while others have gone for more comprehensive approaches and included virtually all known LSD-causing genes [42,45]. Some authors have even went a step further and developed an investigational panel including a large number of genes from the autophagy-lysosomal pathway (ALP), regardless of their known involvement with genetic disease [46,47]. Many other approaches have been used to provide MGT to LSD patients, from commercially available panels to WES and WGS [39,43,46,49,50,51]. Whatever the approach, the efficacy of NGS as a tool kit to detect DNA sequence variations underlying LSDs always seemed obvious. In that sense, our report adds up to the bibliography on the subject, further supporting the utility of using NGS for LSD diagnosis, either as a first- or second-tier test.

Interestingly, while the number of reports on the use of NGS-based approaches for LSD MGT is quite substantial, there are significant methodological differences between the different studies, which we would like to briefly discuss. Concerning targeted panel design, some authors have gone for really small and well-focused panels, whose major purpose was to address LSDs with available therapeutics, or screen for the most common LSDs in order to refer patients for treatment as quickly as possible. The low number of genes contributes to assure great depth of coverage and fast interpretation, while lowering the risk of finding numerous variants of unknown significance (VUS). This was the chosen route for some labs, especially those belonging to reference centers on rare diseases. One perfect example of this sort of approach was that published by Rojas Málaga and co-workers, where the authors designed two independent panels for the simultaneous testing of 11 LSD-related genes based on the criteria of overlapping clinical manifestations [43]. Other authors opted for a much less conservative approach, including numerous genes, which are not known to cause genetic disease whenever mutated. The best example of one such panel in the LSD field is probably “Lysoplex,” a targeted NGS approach for the simultaneous sequencing of 891 genes involved in lysosomal, endocytic, and autophagic pathways [46,47]. This is a well-known panel in the field, based on a Haloplex enrichment protocol. While holding a number of advantages when compared to WES and WGS approaches, with higher target reproducibility, specificity, depth, and sensitivity, it still comprehends an extremely large number of genes, which strongly decreases the speed analysis. Furthermore, the risk of finding numerous VUS is also significantly increased. With a huge number of genes, which have not yet been associated with human Mendelian diseases (751 out of 891) included in the screening, variant interpretation may be much more difficult. As a consequence, it may not be the panel of choice for clinical researchers who ask for fast and cost-effective MGT. Still, it does hold an enormous investigational potential, providing basic researchers with a powerful tool to study the cell biological relevance of protein variants in the ALP pathway.

Here, we chose to design a panel whose rationale lies somehow in between these two extremes. It is extensive enough to include not only the currently known LSD-related genes, but also some genes which are not LSD-related but whose dysfunction causes diseases with overlapping clinical manifestations. Finally, it also accommodates a few other genes, which are not disease-related but encode for particularly relevant ALP proteins. Nevertheless, it mostly relies on disease-related genes (67 out of 85), thus allowing for a relatively straightforward interpretation of the results while holding potential to molecularly characterize patients suffering from virtually every LSD. By accommodating a number of non-disease related genes, it also allows for the identification of novel loss-of-function variants on those genes, which may trigger additional investigations and ultimately extend the catalog of Mendelian inheritance in man. Altogether, this is a robust and cost-effective panel, which allows for the simultaneous screening of virtually all LSDs while holding potential to discover novel disease genes without losing coverage.

In general, NGS technologies hold a huge potential as MGT tools, especially for diseases that are traditionally considered monogenic such as the ones here addressed. With one single run, it is now possible to provide an accurate molecular diagnosis for a growing number of patients. Nevertheless, it is also responsible for the identification of a significant number of VUS. Ideally, functional studies would be the ultimate way to address this issue, by experimentally assessing the overall consequences of each novel variant detected at different levels. Still, functional studies on patients’ samples or, alternatively, in vitro mutagenesis with subsequent expression of mutant proteins, are expensive, time-consuming, and quite laborious tasks. Thus, functional studies may hardly raise up as the front-line approach for pathogenicity assessment in the context of MGT for diagnostic purposes.

In this context, in silico predictors are clearly gaining momentum. Fortunately, the different bioinformatic platforms that offer deleteriousness-prediction scores are evolving as fast as the overall sequencing technology, and incorporating more and more data, in order to become ever more accurate. Still, we chose to add yet another level of in silico analysis to our MGT algorithm, by modeling each amino acid substitution into its three-dimensional protein structure and carefully comparing it with similar substitutions in its surroundings (or even in the same residue). By doing so, we got a much clearer understanding on how a specific substitution could affect protein structure and/or function. Importantly, this sort of studies, while being a valuable option for immediate guidance regarding patient care and counseling, are relatively fast and inexpensive. In fact, attentive in silico analysis may confer an indication of prognosis value to the genotyping results [34].

In this particular set of patients, in silico analysis of the novel missense variants was relatively straightforward, with the pathogenic effect of both variants (p.Trp287Arg in the *GLA* gene and p.Gly455Arg in the *MFSD8* gene) offering no doubts on their deleterious effect over protein structure. Still, this may not always be the case. In fact, structural-based prediction tools may sometimes be unable to accurately predict the effect of a particular variant due to a lack of availability of known homologous structures. For those cases, functional studies are mandatory.

Overall, our findings concerning the Tunisian samples here analyzed are also very relevant, as they sum up to the overall knowledge on the molecular basis of this group of diseases in a population where their incidence is probably underestimated. It is also worth mentioning the high levels of consanguinity observed. Indeed, the overall level of homozygosity found in the Tunisian patients here analyzed is remarkable, with each family presenting its own molecular defect. Altogether, our results contribute to the broad understanding of the molecular basis, enzymatic defects and clinical manifestations of LSDs in Tunisia, further supporting previous reports on the high impact of inbreeding and regional endogamy on the occurrence of autosomal recessive disorders in that country. Hopefully, these results will not only contribute to improve genetic counseling for affected families, allowing carrier detection and prenatal molecular diagnosis, but also to highlight the need for reinforced and continuous information of general public and health professionals on the potential negative medical impact of intra-family marriages, particularly in Northern Africa, the Middle East, and South Asia [52].

## 4. Materials and Methods

### 4.1. Sample/Subjects

Our sample included 23 individuals from different origins: 13 from Portugal, seven from Tunisia, two from India, and one from Cape Verde. Subjects presented with variable degrees of suspicion of LSDs, depending on their clinical and/or biochemical features. The studies were conducted in agreement with the Declaration of Helsinki and submitted to the Ethics Committee of Instituto Nacional de Saúde Dr. Ricardo Jorge (2016DGH1312), where biological samples were obtained.

### 4.2. Next Generation Sequencing

Briefly, genomic DNA was automated extracted and purified from peripheral blood on a BioRobot EZ1 instrument (QIAGEN, Germantown, MD, USA) using the EZ1 DNA Blood 350 µl Kit (QIAGEN, Germantown, MD, USA). We used 50 ng for the library preparation according to the manufacture’s protocol (Sureselect, Agilent Technologies, Santa Clara, CA, USA). We sequenced a custom panel composed of 85 genes (exons and flanking regions). The custom-panel design was prepared using Sure Design software (Agilent Technologies, Santa Clara, CA, USA) for the Targeted Enrichment System and Illumina platform based on the last genome build available (*H. sapiens*, hg19, GRCh37, February 2009) and by selecting for a 150 base-pair read length (based on RefSeq database). The NGS protocol was performed as previously described [53]. The 85 genes panel includes 65 genes encoding lysosomal proteins and 20 genes coding for non-lysosomal proteins. Overall, those genes may be clustered in the following way, according to their involvement in disease: 54 LSDs genes, 13 genes associated with other diseases with overlapping phenotypes, and 18 genes not yet associated with disease but whose impaired function could impact lysosomal homeostasis (Figure 1).

### 4.3. NGS Data Analysis

The MiSeq Reporter software (Illumina, San Diego, CA, USA) was used for sample demultiplexing and FASTQ file generation. Alignment and variant calling were performed using Surecall (Agilent Technologies, Santa Clara, CA, USA). An evenly distributed mean depth of coverage for the panel was achieved and a mean of 98.4% targeted bases were covered at least 100x. The panel design was done in order to have 10 bp upstream and downstream of the exon with almost 100% of coverage. The exception is for pseudogenes. The average read depth in analyzable target regions is 281x. The NGS-data analysis pipeline includes the variants annotation software: wANNOWAR (http://wannovar.wglab.org/). This tool can generate several different types of deleteriousness-prediction scores that take into consideration the evolutionary conservation (such as FATHMM, Mutation Assessor, SIFT) and protein structure function (such as Mutation Taster and Polyphen-2). Variants were filtered according with these in silico predictions. Additionally, the variants with a minor allele frequency (MAF) >1% in the 1000 Genomes Project and Exome Variant Server were excluded. Whenever a novel amino acid substitution was considered as potentially pathogenic on the basis of wANNOVAR annotation and database-screen for frequencies <1%, that substitution was individually mapped into its three-dimensional protein model using the PyMOL tool. Briefly, the three-dimensional structure of the proteins under analysis was obtained from the Research Collaboratory for Structural Bioinformatics (RCSB) Protein Data Bank (PDB; https://www.rcsb.org/); IDs: 1R46 and 3WDO for GLA and MFSD8, respectively. While the X-ray structure of GLA has already been determined [26], the same does not apply to MFSD8. Therefore, we used homology modelling to obtain an in silico model of the overall protein, using as template the structure of *E. coli* YajR transporter reported by Jiang et al. in 2013 [54]. In silico mutagenesis was performed with SWISS-MODEL (https://swissmodel.expasy.org/) by feeding the automated protein structure homology-modelling server with the mutated protein sequence while using the respective wt PDB structure as a reference. Image acquisition and structural analyses were performed with PyMOL. Additional information on those proteins was collected from UniProt (http://www.uniprot.org/), a free online collection of literature-based expert curated annotations on individual protein sequences; IDs: P06280 and Q8NHS3 for GLA and MFSD8, respectively [55].

### 4.4. Sanger Sequencing Analysis

Specific primers were designed (available upon request) to confirm the candidate variants found in the patients under study/analysis and segregation was tested in the appropriate family members (when available). Also, inadequate coverage regions were identified and completed by Sanger sequencing (namely on patient P6). The PCR products were purified using illustra ExoStar™ 1-Step (GE Healthcare Life Sciences, Buckinghamshire, UK), and the sequencing reactions were performed using a BigDye Terminator v3.1 Cycle Sequencing Kit (Applied Biosystems, Foster City, CA, USA). The analysis was performed using an ABI PRISM^®^ 3100 Genetic Analyzer (Applied Biosystems, Foster City, CA, USA). Results were analyzed with the sequence analysis software FinchTV, versions 1.3.1 and 1.4.0.

## 5. Conclusions

In this study, we applied a targeted NGS approach to screen 23 patients with variable clinical suspicion of LSDs for variants in genes involved in lysosomal function. Briefly, we succeeded in the molecular diagnosis of nine of those patients. It is worth mentioning that molecular characterization was relatively straightforward for all the patients with a high suspicion index for LSD, thus demonstrating the potential of our NGS-panel as first-line MGT approach, instead of studying particular sets of genes either by the laborious and time-consuming classical methods or even using other NGS panels with a much more limited number of genes, which have been designed by different teams, based on the criteria of overlapping clinical manifestations [42]. With a single panel, we are able to screen for all known LSDs and a number of related disorders. In fact, this extended panel allows us to gather the necessary number of samples for a single run in a much more efficacious way: targeted panels based solely on the strong phenotypic overlap require much more strict patient selection criteria for each run. In small countries like ours this may either result in a delay on the analysis (if the laboratory decides to wait for the proper number of samples to perform a single run) or in a suboptimal use of flow-cells and overall NGS reagents (if the laboratory chooses to perform the run without filling a minimum/adequate number of samples).

The application of this NGS panel allowed us to identify a very rare form of GM2A-gangliosidosis and a NCL subtype in a single run. In the case of these particular diseases, for which the clinical and biochemical features overlap with other disorders, the NGS technology is especially useful. The classic molecular studies consisted of time-consuming and expensive genetic studies. In fact, the custom-made targeted panel sequencing allows the screening of several patients and many genes with relatively easy and manageable interpretation of post sequencing data.

For the uncharacterized patients (Table 1), other NGS approaches such as WES or WGS should be considered. Nevertheless, we should also bear in mind, when analyzing the overall MGT results, that our set of patients gathered samples with variable suspicion index for LSD. In fact, some of the individuals we screened had a general clinical picture, which was compatible with LSD but overlapped with other pathologies, namely neurodegenerative diseases. Additionally, there were also cases from whom we had no access to clinical data (see Supplementary Table S1). Therefore, most probably the genetic defects underlying pathology in those individuals are not lysosomal-related.

Overall, this study highlights the potential of an expanded/comprehensive NGS targeted panel as a first-tier MGT for LSDs. Ultimately, the generalized use of NGS approaches to screen for LSD in individuals who present with compatible clinical features, will allow for a faster and more accurate diagnosis of several LSDs and contribute to broaden our view on the genetic and mechanistic complexity, which underlies these disorders. In fact, even though LSDs, like many other inherited errors of metabolism, have always been classified as monogenic diseases, data is accumulating on the existence of complex patterns of inheritance, including synergistic heterozygosity, in this sort of disease. In fact, the existence of a multigenic inheritance pattern in which pathogenic variants in multiple genes in a metabolic pathway lead to sufficient disruption of flux through the pathway, mimicking a monogenic disorder caused by homozygous defects in one gene in that pathway, has already been realized for a number of inherited metabolic disorders. In addition, widespread adoption of WES and WGS in medical genetics has led to the realization that individual patients with apparently hybrid phenotypes can have mutations in more than one gene, leading to a mixed genetic disorder [56]. It has long been known that even diseases caused by single-gene pathogenic variants can display substantial phenotypic variability, which may be due to genetic, environmental, or epigenetic modifiers. The presence of modifier genes, for example, that may be involved in determining disease severity is one of the phenomena that may help elucidate phenotypic variability between patients with similar LSD-causing variants. The increasing use of high-throughput sequencing technologies will definitely contribute to further document and understand this phenomenon. It also sums up to the previously unexpected complexity of these so-called monogenic diseases. Altogether, these observations set a trend toward more accurate genetic diagnosis and treatment, which will definitely place LSDs on the leading edge of precision medicine as complex genetic traits.

## Figures and Tables

**Table 1:** Molecular genetic testing (MGT) results for patients shown to harbor LSD-causing pathogenic variants. Whenever clinical and/or biochemical data were available, that information was also included.

Patient	Molecular Diagnosis (#MIM)	Gene (RefSeq)	cDNA	Protein	Variant Type	In Silico Predictors #	Reference	Clinical Data (When Available)/Clinical Suspicion	Biochemical Phenotype	Origin
P1	Gaucher type I (#230800)	*GBA* NM_001005741.2	c. 1448T>C/c. 1448T>C	p.Leu483Pro/p.Leu483Pro	Missense	Pathogenic	[15]	At nine months: mild hepatomegaly; exuberant splenomegaly; feeding difficulties and dysphagia; bilateral convergent strabismus; marked axial hypotonia; poor facial mimic; global psychomotor development delay; cardiomegaly with dilatation of the left cavities; interstitial lung disease with multiple recurrent infections including aspiration pneumonia; three cardiac arrest events. Cerebral MRI showed supratentorial periventricular white matter alterations of tegmentum pontis and dentate nucleus, all aspects which are compatible with central nervous involvement in the context of Gaucher disease	Low GCase levels in skin fibroblasts	Cape Verdean
P2	Fabry disease (#301500)	*GLA* NM_000169	c.859T>C	p.Trp287Arg	Missense	Pathogenic	Novel	Fabry disease	-	Tunisian
P3	Pycnodysostosis (265800)	*CTSK* NM_000396.3	c.436G>C/c.436G>C	p.Gly146Arg/p.Gly146Arg	Missense	Pathogenic	[16]	Pyknodysostosis	-	Tunisian
P4	Mucolipidosis type II (#252500)	*GNPTAB* NM_024312.4	c.571G>A/c.571G>A	p.Val191Ile/p.Val191Ile	Missense	Mostly pathogenic	[17]	Mucolipidosis type II	-	Indian
P5	CLN7 (#610951)	*MFSD8* NM_152778.2	c.1363G>C/c.1363G>C	p.Gly455Arg/p.Gly455Arg	Missense	Pathogenic	Novel	Neuronal ceroid lipofuscinosis	-	Tunisian
P6	Krabbe disease (#245200)	*GALC* NM_000153.3	c.613_617del/c.613_617del	p.Tyr205Ter/p.Tyr205Ter	Deletion	NA	Novel	Krabbe disease	-	Tunisian
P7	GM2A-gangliosidosis AB-variant (#272750)	*GM2A* NM_000405.4	c.312del/c.312del	p.Gly104Gly fsTer14/p.Gly104Gly fsTer14	Deletion	NA	Novel	Neurodegenerative LSD (not specified)	-	Tunisian
P10	MPSIIIB (#252920)	*NAGLU* NM_000263.3	c.934G>A/c.934G>A	p.Asp312Asn/p.Asp312Asn	Missense	Pathogenic	[18]	Mucopolysaccharidosis	-	Tunisian
P13	Alpha-mannosidosis (#248500)	*MAN2B1* NM_000528.3	c.2402dup/c.2402dup	p.Ser802Gln fsTer21/p.Ser802Gln fsTer21	Duplication	Pathogenic	[19]	Alpha-mannosidosis	-	Tunisian

# In silico predictions for novel missense variants relied on the software wANNOVAR, which takes into account the results from a number of well-known algorithms on the phenotypic consequences of amino acid substitutions: FATHMM [20], SIFT [21], Mutation Taster [22], and PolyPhen 2 [23].

## Supplementary Materials

The following are available online at https://www.mdpi.com/1422-0067/21/17/6355/s1. Figure S1. Three-dimensional structure representation of NAGLU using PyMol. Stereo figure showing three-dimensional structure of the NAGLU monomer with the amino acid position 312 (wild type: Asp312; mutated Asn312) highlighted; Table S1. Diagnostic suspicions accompanied by the general clinical picture and/or biochemical phenotype of the remaining patients. Data on the patients’ origin is also presented. -: not available.

## Abbreviations

CTSK	cathepsin K
GCase	β-glucocerebrosidase
GD	Gaucher disease
GLA	α-galactosidase
GM2AP	GM2 activator protein
ID	intellectual disability
LAMAN	α-mannosidase (also known as MAN2B1)
LSDs	Lysosomal Storage Diseases
MAF	minor allele frequency
MFSD8	Major Facilitator Superfamily Domain Containing 8
MGT	molecular genetic testing
MPS	Mucopolysaccharidosis
NAGLU	α-N-acetylglucosaminidase
NCL	neuronal ceroid lipofuscinosis
NGS	Next-generation sequencing
NMD	nonsense mediated mRNA decay
PTC	premature termination codon
SNP	single-nucleotide polymorphism
VUS	variants of unknown significance
WES	whole exome sequencing
WGS	whole genome sequencing
