# Supplementary material for: Assessing Lysosomal Disorders in the NGS Era: Identification of Novel Rare Variants
Source: Int J Mol Sci. 2020 Sep 1;21(17):6355. doi: 10.3390/ijms21176355 (PMC7503609; doi:10.3390/ijms21176355)
Supplement: Supplementary file 1 [file ijms-21-06355-s001.zip › Supplementary Figure 1.docx]

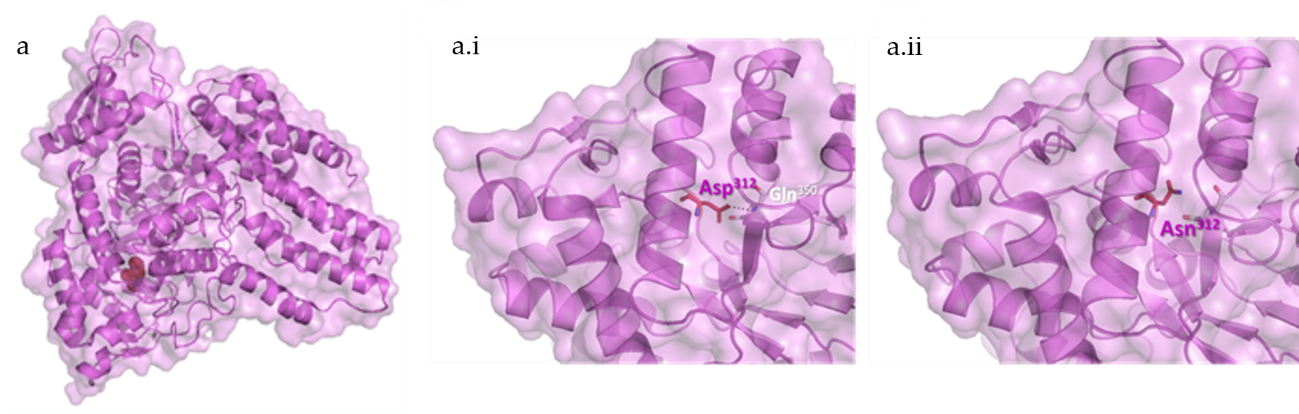


**Supplementary Figure 1.** Three-dimensional structure representation of NAGLU using PyMol. β-strands, helices and coils indicate the secondary structure elements that form the scaffold for the interacting residues. Relevant side chains that interact with the residues of interest are depicted and labelled. **(a)** Crystal structure of the human N-acetyl-α-glucosaminidase (NAGLU) shown in ribbon representation, according to the X-ray diffraction studies by Birrane and co-workers (Protein Data Bank accession code =4XWH; reported resolution for this entry = 2.32 Å). The three-dimensional structure analyzed refers to the mature protein, which has 23 amino acids less than the coded NAGLU (signal peptide). Relevant side chains that interact with the residue of interest are depicted and labelled. Stereo ﬁgure showing three-dimensional structure of the NAGLU monomer with the amino acid position 312 (wild type: Asp^312^) highlighted. (ai) Zoomed in view of the wild type Asp^312^; (aii) mutated Asn^312^.
