# Supplementary material for: Assessing Lysosomal Disorders in the NGS Era: Identification of Novel Rare Variants
Source: Int J Mol Sci. 2020 Sep 1;21(17):6355. doi: 10.3390/ijms21176355 (PMC7503609; doi:10.3390/ijms21176355)
Supplement: Supplementary file 1 [file ijms-21-06355-s001.zip › Supplementary Table 1_19.08.2020.docx]

**Supplementary Table 1.**

Diagnostic suspicions accompanied by the general clinical picture and/or biochemical phenotype of the remaining patients. Data on the patients’ origin is also presented.

| **Patient** | **Clinical data** (when available)**/**  **Clinical suspicion** | **Biochemical phenotype** | **Origin** |
| --- | --- | --- | --- |
|  |  |  |  |
| P8 | Consanguineous parents; thickening of thights and knee, seizures, development delay; severe hyperpigmentation, coarse face; anterior ends of the ribs are flared; death at 3 months old | Elevated levels of lysosomal enzymes in plasma | Indian |
| P9 | 50 years with Parkinsonian syndrome; hypotonia; hypophagia/  Niemann-Pick type C suspicion | Positive filipin staining (less intense than a typical NPC) | Portuguese |
| P11 | General suspicion of LSD | - | Portuguese |
| P12 | Clinical picture of Metachromatic Leukodystrophy | - | Portuguese |
| P14 | Newborn presenting coarse fácies, short stature and skeletal alterations | - | Portuguese |
| P15 | Coarse facies, limb deformities, recurrent hernia, development delay, macrocephaly, MRI – loss white matter | Alteration of GAGs levels, normal lysosomal enzymes | Portuguese |
| P16 | Coarse facies, short stature, development delay | Normal lysosomal enzymes | Portuguese |
| P17 | Child with 6 months, cardiorespiratory arrest at 1 months (resulting in neurologic changes), coarse facies | Alteration of GAG levels | Portuguese |
| P18 | Trachemalagia at birth, bilateral cryptorchidism (solved) umbilical hernia (solved), eye disease, cerebral NMR: macrocrania and frontal bossing, normal cognitive level | Negative GAGs, normal lysosomal enzymes | Portuguese |
| P19 | 17-year-old boy with treatment-resistant psychosis  liver volume at the upper limit of normal  consanguineous maternal grandparents  normal biochemical study  mutation in the ATP7B gene in heterozygote (Wilson disease)  second cousin in the maternal line with wilson's disease | - | Portuguese |
| P20 | 22 years old with neurologic picture | - | Portuguese |
| P21 | - | - | Portuguese |
| P22 | 10 months with chronic anemia, hipotonia | - | Portuguese |
| P23 | Coarse facies with anteverted nostrils, arched eyebrows | Negative GAGs, normal lysosomal enzymes | Portuguese |
